# Urinalysis in Great Dane Puppies from Birth to 28 Days of Age

**DOI:** 10.3390/ani10040636

**Published:** 2020-04-07

**Authors:** Monica Melandri, Maria Cristina Veronesi, Salvatore Alonge

**Affiliations:** 1 Società Veterinaria “Il Melograno” Srl, Sesto Calende, 21018 Varese, Italy; drsalvatorealonge@gmail.com; 2 Department of Veterinary Medicine, Università degli Studi di Milano, 20100 Milano, Italy; maria.veronesi@unimi.it

**Keywords:** dipstick, dog, neonates, newborns, refractometer, urinalysis

## Abstract

**Simple Summary:**

Still, nowadays, small animal perinatology is quite an unknown field of veterinary medicine. To help decrease the high mortality rates reported for canine newborns, veterinary practitioners should become more aware of neonatal physiology and pathology, focusing their attention on metabolic balance. It is impossible to discriminate healthy puppies from pathological littermates in the absence of reference ranges for specific parameters, which are obviously different from those of adults. Since blood samples are more difficult and dangerous to collect from newborns, practitioners could rely on urinary samples, easily gathered by the stimulation of the somato-vesical spinal reflex. The present research, based on 624 urine samples, offers results that can be used as references for the first-line evaluation of newborn Great Danes by dipstick rapid urinalysis. In healthy puppies, specific gravity and pH vary from birth to 28 days of age, while glucosuria and proteinuria are never reported. The present results, derived from a sample of single-breed puppies, can represent the starting point for a further multi-breed evaluation.

**Abstract:**

Urinalysis, a common test in infants, could represent a suitable non-invasive clinical tool in puppies. In dog neonates, urine is easily collected by stimulating the somato-vesccal reflex. Information on urine characteristics during the neonatal period is missing. Beside instrumental laboratory analyses, the dipstick was proven useful for rapid urinalysis to evaluate specific gravity (SG), pH, leukocytes, nitrites, glucose, proteins, ketones, urobilinogen, bilirubin, and blood. The present study aimed to describe urinalysis features by the dipstick test and refractometer along the neonatal period. Urine samples (n = 624) were collected by manual stimulation from 48 healthy Great Danes, daily from birth to seven days, then twice a week until 28 days, to assess age-related changes (ANOVA, *p* < 0.05) and the possible effects of gender and litter (T-test, *p* < 0.05). The SG and pH significantly changed during the neonatal period. Other parameters did not vary significantly in relation to age. No significant differences were observed either among litters or between genders. The present study confirmed that canine kidneys are able to concentrate urine from the second week of age when the urinary SG started to be similar to adults, while pH still increased towards the typical values of adults at 28 days. Significant glucosuria and proteinuria were never detected. Dipstick urinalysis represents a useful first-line complementary tool in newborns clinical examination, providing information about systemic homeostasis.

## 1. Introduction

In canine medicine, little is known about perinatology, though a deeper knowledge would be desirable in order to reduce morbidity and mortality rates, which are as high as 10–20%, possibly reaching peaks of 40% [1]. Different illnesses can be responsible for neonatal mortality; some of them are easily diagnosed, such as congenital, infectious, and parasitic diseases. Diagnostic tools and potentials for homeostatic abnormalities (i.e., hydration levels, hyper- and hypoglycemia, fasting, hyper- and hypothermia) have not been investigated deeply, even if they can strongly be suspected when morbidity and mortality rates are high [2]. The homeostatic balance is essential in adults and even more in neonates, whose self-regulation turns out to be much more fleeting.

The clinical management of ill canine neonates still represents a challenge because of the limited availability of diagnostic tools; in many instances, normal (physiologic) reference data are missing, too.

Few studies looked for markers of neonatal homeostasis, but they still cannot be widely applied in everyday veterinary clinical practice: lactate, glucose and beta-hydroxybutyrate levels were correlated to neonatal mortality [3]; the Combur test analysis on glucose and ketones in the fetal fluids collected at C-section was described [4]; the composition of the fetal fluids at C-section was linked to neonatal mortality and viability [5].

The assessment of hemato-biochemical parameters, representing the first gold standard screening diagnostic tool in young and adult dogs, cannot routinely be applied in canine neonatology, because of both the technical difficulties to obtain high-volume and high-quality samples and the invasiveness of sample collection procedures due to the small size and the characteristic fragile veins of the newborn puppy. As a result, small animal neonatologists cannot rely on hemato-biochemical features for diagnostic/prognostic reference values.

Therefore, the assessment of urinary parameters, that represent the most common biochemical test in infancy and early childhood in human medicine [6], could represent a suitable and non-invasive first-line diagnostic tool also in canine neonatology [7].

Even if micturition can easily be elicited in neonates through a somato-vesical spinal reflex induced by perineal stimulation up to 3–5 weeks after birth [8], urinalysis in neonatal puppies has not been completely elucidated, yet, and reference parameters are not provided. Some authors investigated the relationship between age and renal function to describe the maturation of canine kidneys by sophisticated measurements of tubular or glomerular function, in a small number of puppies belonging to an experimental Beagle colony during pediatric age [9]. Subsequently, Faulks and Lane [7] compared results of urinalysis in healthy, random-source dogs, casually sampled just once at the age of 0–24 weeks. The urinalysis is depicted as a key component in the diagnostic evaluation of puppies. The recognition of abnormal urinalysis results depends on a definition of normal parameters or reference ranges, but, to the authors’ knowledge, data about the urinalysis changes occurring along the neonatal period in dogs are lacking.

Urinalysis can be performed on voided samples or on urine collected by urethral catheterization or by cystocentesis [10]. Concerning neonates, voided samples are satisfactory for a routine urinalysis screening, especially for assessing urine concentration and metabolic parameters, such as pH, glucosuria, bilirubinuria, and ketonuria [7,8], but they are not suitable for urinary bacterial cultures. For this purpose, cystocentesis is required [11], but it is too invasive in newborns due to the risk of bladder injuries, as urethral catheterization is [1].

Therefore, voided urine samples obtained through manually-induced somato-vesical spinal reflex, represent the best option to perform the first-line urinalysis in canine neonates, mainly in clinical settings. Traditional laboratory urinalysis, including physico-chemical, microbiological, and sediment examinations, usually require large volume samples, difficult to obtain with a single collection from newborn dogs. Moreover, in many instances, analysis results are available hours or days after the urine collection, impairing the necessary prompt diagnosis for the clinical management of newborn dogs, in which changes in the general conditions can occur suddenly.

As a consequence, in newborn dogs, an alternative, low-volume, quick and basic urine assessment tool is necessary. Urinalysis dipstick tests are largely used as a screening tool for the basic assessment of patients both in human and veterinary medicine [12,13]. Dipstick urinalysis is easily performable under every condition, is a cheap and repeatable diagnostic test, providing immediate results. Moreover, as reported by Balogh et al. [4], a drop of urine can be put on each field of the strip to obtain reliable results using small volumes of urine. Thanks to their advantages, dipstick tests are, therefore, widespread in veterinary companion animals’ clinical practice. Urinalysis dipsticks were reported to be useful also in canine perinatology. In a first study, Faulks and Lane [7] reported data on urinalysis in 0–24-week-old puppies, while more recently, Balogh et al. [4] used dipstick to differentiate maternal urines from amniotic and allantoic fluids at the beginning of parturition, in dogs. On the other hand, dipstick tests do not analyze either sediment or bacteria. Besides the known advantages of this diagnostic tool, the reading system, which is based on a colorimetric scale that can lead to subjective mistakes in the final interpretation of the parameters, represents a disadvantage.

Dipstick tests for urinalysis give information on specific gravity (SG), pH, leukocytes, nitrites, glucose, proteins, ketones, urobilinogen, bilirubin, and blood.

Because of the multi-systemic immaturity at birth and along the neonatal period, newborn puppies are prone to develop dehydration. Urinary SG is recognized as a simple, inexpensive, and useful clinical indicator of the hydration status, polyuric disorders, and of the renal concentrating or diluting function [14]. In combination with other components of the urinalysis, such as proteins and glucose, the urinary SG is also useful for estimating the severity of loss of substances in the urine [14]. It is generally recommended to determine urinary SG by means of a refractometer, but it can be also measured by dipstick tests [10]. 

Urinary pH in different animal species is linked to alimentary habits; in carnivores, including dogs, it normally ranges between 6 and 6.5, being slightly acid [15]. The pH represents the content of hydrogen ions in body fluids, whose balance in different compartments is regulated from the kidneys and the lungs. In the case of acute or chronic acidosis or alkalosis, the renal excretion of hydrogen ions and bicarbonates is altered in order to keep the neutral pH of blood, essential for survival, influencing in the meanwhile the urinary pH. Thus, the urinary pH is influenced by alterations in systemic homeostasis [15]. Considering the delivery day, a mild to severe combined respiratory-metabolic acidosis, diagnosed by blood gas and acid-base status, is observed in almost every newborn puppy [16]; therefore, the blood and urinary pH values are low at birth. In neonates, the renal function is still immature, especially the tubular one, also in charge of the secretion and reabsorption of bicarbonates as a buffer system for blood pH regulation in adults [17,18]. As a result of the lack in this function and in order to keep the blood alkaline reserve, the neonatal glomerulus filters’ hydrogen ions are not reabsorbed by the tubule. As a consequence, the urinary pH of neonates is lower than that of adults, and it reaches normality, thanks to renal maturation with increasing age. 

Glucose can appear in urine if glycemia exceeds the renal threshold or if the renal tubular reabsorption is decreased. Because of the immature tubular function, normoglycemic glucosuria has been expected in normal young puppies [12,19].

Proteinuria, i.e., the excessive elimination of proteins in the urine, is a feature of tubular or glomerular dysfunction. According to the literature, marked but physiological proteinuria during the first two weeks of life is expected [20]. Some authors previously reported that the total daily amount of proteins excreted by 2-month-old puppies (6 ± 1.9 mg/kg of body weight, BW) is lower than that excreted by adult dogs (48 ± 68.4 mg/kg BW) [21]. Due to the partial capacity of the glomerular system to filter and of the tubules to reabsorb urine, proteinuria, and aminoaciduria have been described in neonates whose renal development is incomplete until 2–3 weeks after birth [22,23,24]. 

Since it has been proven that breed can be a factor affecting normal hemato-biochemical parameters in adult dogs [25,26], it could be supposed that breed can affect urinary parameters, too. However, to the authors’ knowledge, this has not been investigated yet, neither in adults nor in young or neonate puppies. In the authors’ opinion, special attention should be devoted when canine breeds predisposed to congenital renal defects or malformations are concerned, such as Alport’s syndrome [27], necessarily affecting urinary parameters.

Because of all the above-mentioned reasons, the aim of the present study was to describe the features of urinalysis in healthy newborn dogs belonging to a single breed, from birth to 28 days of age, by a commercial urinalysis dipstick test, performable in routine veterinary practical settings.

## 2. Materials and Methods

### 2.1. Animals

In the present study, six Great Dane litters born by 2–5-year-old bitches of first and second parity, belonging to a single kennel, were enrolled. Great Danes were chosen because, to the authors’ knowledge, no hereditary renal defects have been reported for this breed in today’s literature. 

The bitches were healthy [28], their body condition score ranging between 2.5 and 3 on a scale of 5 [29]; they were regularly vaccinated and submitted to parasite prophylaxis, fully evaluated from the beginning of the estrous cycle, monitored by blood progesterone concentration and mated once two days after the estimated ovulation time [30] with males of proven fertility [31]. Pregnancy diagnosis was performed by ultrasound 20–22 days after mating, and from that time, all the bitches were fed with commercial feed, specifically for pregnant female dogs. Pregnant bitches were monitored throughout the whole pregnancy, and only uncomplicated pregnancies were considered. For all the patients enrolled in the study, C-section was planned in view of the health of the mother and the puppies, due to previous history or expected troubles at parturition [32]. Elective Cesarean section was planned based on several parameters, including blood progesterone concentration during estrous and at the end of pregnancy, parturition date forecast by fetal ultrasonographic biometric parameter records, such as the inner chorionic cavity and the biparietal diameter, evaluated in early and late pregnancy, respectively [33,34], fetal well-being according to the fetal/maternal heart rate ratio during pregnancy, and fetal heart rate at term [35]. Concerning the anesthetic protocol for the C-section, no premedication was given, and general anesthesia was induced with alfaxalone 2 mg/kg IV, titrated to effect for oro-tracheal intubation, while maintenance was achieved with isoflurane in oxygen. Opioids and non-steroidal anti-inflammatory drugs (NSAIDs) were administered to the bitches only after the last puppy extraction [36].

Immediately after birth, puppies were submitted to a neonatal clinical examination. Within 5 min, the viability was assessed by the Apgar score, as reported by Veronesi et al. [2]. Puppies were weighted before suckling, clinically examined to verify the absence of gross physical defects or malformations, and their gender was recorded [37]. Only healthy and viable puppies without clinically evident malformations and with normal body weight at birth (500–750 g) [38,39] were enrolled. All the puppies were exclusively fed by their natural mother until 28 days of age; therefore, the beginning of weaning was delayed after the end of the study. The health status of each puppy was checked daily via clinical examination, coupled to body weight gain measurements until 28 days of age, and puppies having a normal neonatal course, only, were kept in the study.

### 2.2. Ethics

The present study was performed in accordance with the ethical guidelines of the animal welfare committee, and all the procedures were carried out according to the Italian legislation on animal care (DL No. 116, 27/01/1992) and the European Guidelines on Animal Welfare (Directive 2010/63/EU). The present study was carried out on privately owned dogs. The owner signed informed consent to allow the collection of urine at birth and during the neonatal period, as well as the use of all recorded data for research purposes.

### 2.3. Sample Collection and Urinalysis

Urinalysis was performed on voided samples, as already suggested by Faulks and Lane [7]. The first urine sample was collected from each newborn puppy on the day of delivery, after the first suckling, always within 2 h after birth. Because of the recognized possible influence of feeding, diet, and circadian rhythms [40] on water intake and urine SG in dogs, urine samples were collected from the day after birth always at the same time in the morning, daily from two to seven days of age, then twice a week until 28 days of life.

Urines were collected by manual stimulation of the typical neonatal somato-vesical reflex into a sterile plastic vial. They were immediately evaluated by commercial dipsticks (Combur-Test^®^, Roche Diagnostic Limited, Mannheim, Germany) for SG, pH, leukocytes, nitrites, glucose, proteins, ketones, urobilinogen, bilirubin, and blood. Following the technique reported by Balogh et al. [4], a drop of urine was put on each field of the strip and, after dripping, the results were read after 60 seconds for all the parameters, except for leukocytes that were read at 120 seconds. For each parameter, the results were recorded using the visual reference colorimetric scale reported by the company (Table 1). The SG was also evaluated in each sample using a manual refractometer to verify the effectiveness of the dipstick results.

Sample collection and urinalysis were always performed by the same operator for each puppy and at each time point.

Moreover, at one year of age, all the puppies enrolled in the study underwent standard serum hemato-biochemical profile and urinalyses, additional to the clinical exam, in order to confirm that they were healthy and not affected by subclinical renal congenital defects.

### 2.4. Statistical Analysis

All data obtained were reported on an Excel 2010 Office file.

Among the semi-quantitative dipstick parameters, SG and pH were considered as continuous variables, while leukocytes, nitrites, proteins, glucose, ketones, urobilinogen, bilirubin, and blood were considered as ordinal variables.

The normality of the data distribution for all continuous variables was checked by the Shapiro–Wilk test.

The possible changes of urinalysis parameters along with age were assessed by the analysis of variance (ANOVA repeated measures) for continuous normally distributed variables, and the U-Mann–Whitney test, for ordinal variables.

T-test and ANOVA were used to assess statistical differences between males and females and among litters, respectively.

The T-test for paired samples was also performed to evaluate possible differences in SG measured by dipstick or refractometer.

All statistical analysis was performed by the online statistical tools VassarStats: Website for Statistical Computation (http://vassarstats.net, Vassar College, New York, NY, USA) and Social Science Statistics (https://www.socscistatistics.com, Jeremy Stangroom, USA). The probability value of *p* < 0.05 was considered statistically significant.

## 3. Results

### 3.1. Animals

Since all the six bitches were healthy, had a normal pregnancy course, and gave birth to healthy, viable, normal-weighted puppies, without gross physical defects or malformations, all the 48 neonates were first enrolled in the study. After birth, all the puppies showed a normal neonatal course and body weight gain, according to the reference growth curves for the Great Dane breed [38,41]. Therefore, data about urine characteristics were gathered from all the 48 puppies from birth to 28 days of age. Moreover, all the dogs turned out to be healthy at the clinical exam performed at one year of age, when serum hemato-biochemical profiles and urinalysis were within the normal ranges.

### 3.2. Urinalysis

From the 48 puppies, a total of 624 urine samples from birth to 28 days of age were collected and analyzed by dipstick and refractometer. Volume samples ranged between 0.5 and 2.5 mL.

**Figure 1:**
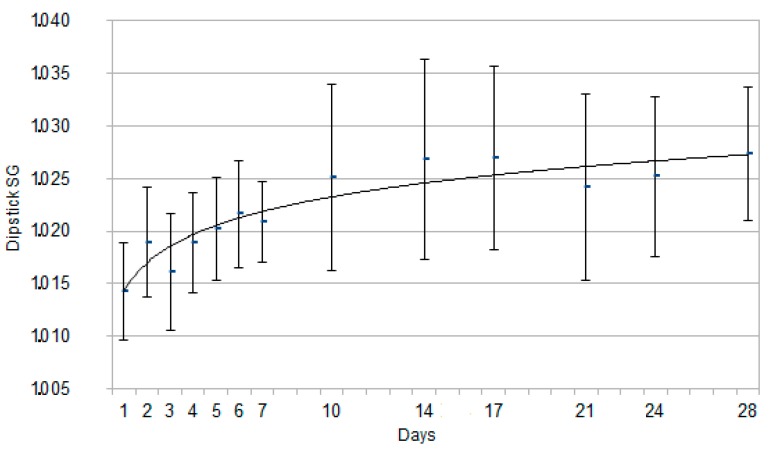
Graph for each time point representing Specific Gravity (SG) measured by dipstick, expressed as mean ± SD, in the 48 Great Dane puppies from birth to 28 days of age. The continuous curve represents the tendency line for this parameter.

**Figure 2:**
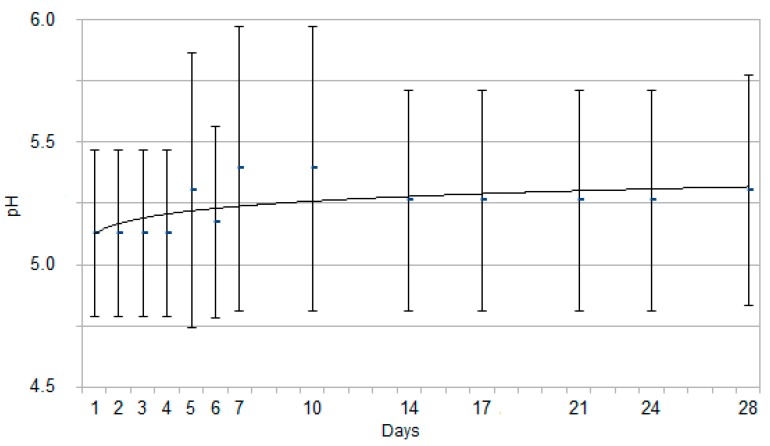
Graph for each time point representing pH, expressed as mean ± SD, in the 48 Great Dane puppies from birth to 28 days of age. The continuous curve represents the tendency line for this parameter.

**Figure 3:**
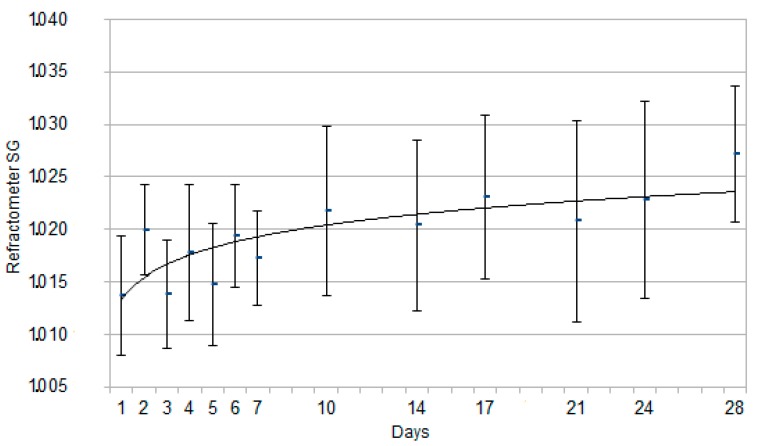
Graph for each time point representing Specific Gravity (SG) recorded by refractometer, expressed as mean ± SD, in the 48 Great Dane puppies from birth to 28 days of age. The continuous curve represents the tendency line for this parameter.

The results for SG and pH by dipstick are reported in Figure 1 and Figure 2, expressed as mean ± standard deviation. The results for SG by refractometer analysis are reported in Figure 3, expressed as mean ± standard deviation. An interpolation line was built, referring to the trend followed by the mean values of each parameter on the study days.

The urinary SG and pH showed statistically significant changes according to newborn puppies’ age, increasing along with the neonatal age. Concerning SG, significant differences from day 1 (1.014 ± 0.005) were identified since day 4 (1.018 ± 0.005), as it can be explained visually by the interpolation line, with an early deflection on day 4. Referring to pH (5.1 ± 0.3), the statistically significant increase was found since day 5 (5.3 ± 0.6), as it can also be inferred by the interpolation line with a late deflection on day 5.

No statistically significant differences were ever observed on SG measured by either dipstick or refractometer (*p* > 0.05).

The results for leukocytes, proteins, and blood are reported in Table 2.

Proteins, leukocytes, and blood did not significantly change in relation to the age of the newborns.

Significant mean values of proteinuria were not observed, although traces (mean corresponding dipstick interval <30 mg/dL) of proteins were found 10 days after birth in 65.2% of neonates.

Except for the first day of life, when leukocytes were absent, a mean of up to 75 leukocytes/µL was found at four days of age in 73.9% of neonates; a mean of 10–25 leukocytes/µL was found in 11.1% of puppies on day 3 and in a range from 4.3% to 26.1% between five and 28 days of age.

Blood was found in almost all the samplings with a mean maximum of 10 erythrocytes/µL, while hemoglobin was found in the first seven days and again at 14 days after birth.

None of the urinary samples reacted for nitrites, glucose, ketones, urobilinogen, and bilirubin at any time point.

Finally, no statistically significant differences (*p* > 0.05) concerning urinary parameters were observed either among litters or between males and females.

## 4. Discussion

Only puppies belonging to the same breed were enrolled in the present study, in order to limit possible confounding effects.

The absence of gender effects on the oscillation of urinary parameters is in agreement with the data reported on pigs, in which aquaporin 1–4 and vasopressin V2 receptor expression in the fetal and neonatal kidney during development were not influenced by the gender [42].

The present results prompt to consider SG by dipstick valuable for rapid consultations afield. As no statistically significant differences were found between refractometer and dipstick SG data, dipstick can be considered a good tool afield when the gold standard refractometer [43] is not immediately available.

The mean urinary SG obtained on the day of birth (mean ± SD, 1.014 ± 0.005) was similar to that reported in 11 canine fetuses (1.014) in a previous study [44].

Over the second week of age, the mean urinary SG is approximately 1.025, confirming that puppies aged >3 weeks seem to be able to produce more concentrated urine than immediately after birth, although individual renal function maturation can occur at different times in the postnatal period, so that not all puppies may be able to respond to rapid changes in body water or sodium content [17,22,45].

The urinary SG in newborn puppies was reported to be lower (1.006–1.017) than in adults from birth to four weeks of age, reaching values comparable to adult dogs (>1.030) only after eight weeks of age [46]. In the present study, a significant increase was observed starting from four days after birth (*p* < 0.05)—very low mean values were found only during the first four days of life and showed a continuous increase up to 1.027 at 28 days of age, similarly to the data more recently described by Faulks and Lane [7]. These results confirm that in newborn puppies, renal maturation is a continuous process and that already in the fourth week, the urinary SG is similar to that of adult dogs.

The results from the present study referring to pH are in contrast with previous reports [7]: pH significantly (*p* < 0.05) increased along with age, from 5.1 ± 0.3 (mean ± SD) on the day of birth up to 5.3 ± 0.5 (mean ± SD) at 28 days of life. On the other hand, these data well reflect the immaturity of the tubular renal function in neonates, which is still not able to reabsorb hydrogen ions filtered through the glomerular membrane to maintain the blood alkaline reserve [17,18]. At the end of the study, at 28 days of age, the urinary pH was still lower than normal adult values; it could be inferred that it should probably continue to increase in the following weeks. Literature reports that urinary acidity is enhanced in preterm or suffering puppies [47]; these data could not be compared with present study results because all the puppies enrolled were healthy, viable, and born at the term of normal pregnancy [32].

Even if glucosuria has been reported as a frequent finding in subjects aged ≤8 weeks [46], from the results of the present study, and as already reported by Faulks and Lane [7], no positive reaction to glucose occurred in any healthy puppy, even though the renal function is not completely efficient, as suggested from the urinary SG.

Recent studies in human medicine confirm that neonates have a renal threshold for glucose, similarly to adults, even if kidneys are still partially immature and unable to concentrate. Glucosuria in neonates follows, as in adults, hyperglycemia, or renal damages, both pathological conditions [48]. Hyperglycemia is reported in humans as a common complication of prematurity and is associated with increased mortality [49]. Thus, in canine neonates, too, glucosuria could be expected in premature or ill puppies; it was not observed in subjects enrolled in the present study as they were healthy and at term.

In the present study, a mean significant value for proteinuria was occasionally detected by dipstick urinalysis only at 10 days after birth, with a very low concentration (mean corresponding dipstick interval <30 mg/dL, which corresponds to “traces”), when 65% of the subjects enrolled in the study had a positive reaction to proteins. On all the other sampling days, the mean urinary protein concentration was below such a threshold interval. However, it should be kept in mind that dipstick tests do not take the renal filtration rate into account. The degree of blue staining in the test pad is correlated, in particular, with the strength of the albumin reaction with tetrabromophenol. False-positive findings can arise because of mucus, blood, or highly alkaline (pH > 8) and highly concentrated urine; false negatives occur because of diluted urine [50]. Thus, the low sensibility of the dipstick test in diluted urine, which is typical in neonates having low urinary SG, might explain the absence of proteinuria in the present study. Finally, since some authors reported that proteinuria could be a possible finding in normal newborn puppies up to three weeks of age due to the morphologic and functional immaturity of the kidney [20,22,23,24], further studies are required to depict the cut-off limit to distinguish normal from subclinical/clinical disease conditions.

In the present study, macrohematuria was never observed, even though occult blood and hemoglobin were very often positive on dipstick analysis (Table 2). Transient hematuria is commonly not linked to a pathological relevance. Some differential diagnoses to positive hemoglobin reaction on dipstick test pad could be indicated in myoglobinuria, bilirubinuria, or contamination with oxidizing compounds (e.g., bleach, iodine, peroxidase—from leukocytes or microbes). Thus, sediment examination is mandatory to confirm microhematuria [6,50]. None of the neonates enrolled in this study developed renal or urinary tract diseases consistent with persistent hematuria. Thus, even if it should be confirmed by sediment examination, the transient microhematuria could be considered without clinical relevance, it still remains unclear why many samples were positive for blood [7].

The majority of the samples showed positivity for leukocyte esterase reaction (Table 2). Leukocyturia, the loss of white blood cells in the urine, is mainly associated with urinary tract infections; as an isolated finding, it is not very specific [51]. Also, in human medicine, many authors suggest to perform a microscopic urinalysis, and they still debate the meaning of leukocyte esterase reaction of urinary dipsticks as a fully adequate substitute for microscopy [6,51,52,53,54]. The leukocyte esterase positivity can be caused by the products of leukocyte lysis or other unrelated materials, even when no leukocytes are counted on microscopy [55]. Furthermore, in veterinary medicine, the leukocyte esterase test is not considered reliable in dogs and cats, and its use is controversial also in young and adult subjects [50]. Thus, the positive results for leukocytes obtained in the present study require further investigation to depict their actual value and clinical relevance, since no puppies developed symptomatic urinary tract infections.

Since none of the samples showed a positive reaction to nitrites, ketones, urobilinogen, and bilirubin, it seems possible to suggest that in normal healthy Great Dane puppies, those molecules are absent from birth to 28 days of age and that possible positivity should be considered a necessity for in-depth investigations. Dipstick test only identifies direct bilirubin and some kinds of ketone bodies, namely acetoacetate and acetone, but not beta-hydroxybutyrate, which is responsible for acidosis. As a consequence, the results on ketones must always be interpreted with caution in neonates as well as in adults [56].

## 5. Conclusions

The present study showed that, in healthy newborn single-breed dogs along the first 28 days of life, only urinary SG and pH vary according to the age independently of the gender, while significant glucosuria and proteinuria are almost absent. Being aware of the limited value of dipsticks on the evaluation of the red and white blood cells present in the neonatal urine, the frequent finding of positive reactions despite normal clinical conditions needs further investigations. The Dipstick test urinalysis is therefore useful in clinical settings and provides quick results as a first-line diagnostic tool also in canine neonatology. In ill or critical neonates, it is however recommended to include also a urinary sediment examination in order to better evaluate the finding of hematuria or leukocyturia.

The parameters reported in the present study, obtained in a very wide group of healthy puppies from the same breed, can be considered as preliminary results. Further future investigation on even larger numbers of puppies belonging to several breeds would provide useful reference ranges specifically for newborn puppies.

## Figures and Tables

**Table 1:** Visual reference colorimetric scale corresponding to semi-quantitative results for each urinary parameter, as reported on the Combur-Test^®^ label.

Combur-Test^®^ Parameter	Semi-Quantitative Results Corresponding to the Dipstick Visual Reference Colorimetric Scale						
Specific gravity kg/L	1.000	1.005	1.010	1.015	1.020	1.025	1.030
pH	5	6	7	8	9		
Leukocytes cells/μL	Negative	10–25 (1+)	75 (2+)	500 (3+)			
Nitrites mg/dL	Negative	>0.05 (1+)					
Proteins mg/dL	Negative	30 (1+)	100 (2+)	500 (3+)			
Glucose mg/dL	Negative	50 (1+)	100 (2+)	300 (3+)	1000 (4+)		
Ketones mg/dL	Negative	10 (1+)	50 (2+)	150 (3+)			
Urobilinogen mg/dL	Negative	1 (1+)	4 (2+)	8 (3+)	12 (4+)		
Bilirubin mg/dL	Negative	0.5 (1+)	(2+)	6 (3+)			
Blood erythrocytes/µL	Negative	5–10 (1+)	25 (2+)	50 (3+)	250 (4+)		

**Table 2:** Dipstick parameters recorded as intervals showing positive reaction at any time point during the study (leukocytes, proteins, blood) in the 48 Great Dane puppies from birth to 28 days of age. The number of puppies in each dipstick value-corresponding category is reported for each study day.

Day	Leukocytes Cells/μL				Proteins mg/dL				Blood ery/μL				
Intervals	0	10–25	75	500	0	30	100	500	0	5–10	25	50	250
1	48 *	0	0	0	44 *	4	0	0	24	20 *	4	0	0
2	46 *	2	0	0	42 *	6	0	0	24	14 *	10	0	0
3	29	6 *	13	0	44 *	4	0	0	20	10 *	6	0	12
4	4	6	36 *	2	44 *	4	0	0	18	30 *	0	0	0
5	22	10 *	12	4	44 *	4	0	0	16	18 *	8	4	2
6	16	8 *	16	8	44 *	4	0	0	10	24 *	6	4	4
7	16	14 *	14	4	46 *	2	0	0	12	18 *	6	10	2
10	30	2 *	10	6	18	30 *	0	0	34	4 *	0	6	4
14	30	2 *	10	6	31 *	15	2	0	28 *	4	2	4	0
17	29	1 *	8	10	28 *	20	0	0	39	2 *	2	0	5
21	29	1 *	10	8	38 *	8	2	0	36	6 *	2	0	4
24	28	2 *	4	14	42 *	6	0	0	38	4 *	2	0	4
28	25	3 *	6	14	46 *	2	0	0	38 *	6	0	4	0

The asterisk * indicates the mean corresponding dipstick interval for each parameter on each day.
